# The Microbiome–Genetics Axis in Autism Spectrum Disorders: A Probiotic Perspective

**DOI:** 10.3390/ijms252212407

**Published:** 2024-11-19

**Authors:** Marija Mihailovich, Maja Tolinački, Svetlana Soković Bajić, Sanja Lestarevic, Milica Pejovic-Milovancevic, Nataša Golić

**Affiliations:** 1 Institute of Molecular Genetics and Genetic Engineering (IMGGE), University of Belgrade, 11042 Belgrade, Serbia; maja.tolinacki@imgge.bg.ac.rs (M.T.); svetlana.sokovic@imgge.bg.ac.rs (S.S.B.); natasa.golic@imgge.bg.ac.rs (N.G.); 2 Human Technopole, 20157 Milan, Italy; 3 Institute of Mental Health, 11000 Belgrade, Serbia; sanja.lestarevic@imh.org.rs (S.L.); milica.pejovic@imh.org.rs (M.P.-M.); 4 Faculty of Medicine, University of Belgrade, 11000 Belgrade, Serbia

**Keywords:** probiotics, ASD, autism, clinical trials, microbiota

## Abstract

Autism spectrum disorder (commonly known as autism) is a complex and prevalent neurodevelopmental condition characterized by challenges in social behavior, restricted interests, and repetitive behaviors. It is projected that the annual cost of autism spectrum disorder in the US will reach USD 461 billion by 2025. However, despite being a major public health problem, effective treatment for the underlying symptoms remains elusive. As numerous literature data indicate the role of gut microbiota in autism prognosis, particularly in terms of alleviating gastrointestinal (GI) symptoms, high hopes have been placed on probiotics for autism treatment. Approximately twenty clinical studies have been conducted using single or mixed probiotic cultures. However, unequivocal results on the effect of probiotics on people with autism have not been obtained. The small sample sizes, differences in age of participants, choice of probiotics, dose and duration of treatment, outcome measures, and analytical methods used are largely inconsistent, making it challenging to draw distinctive conclusions. Here, we discuss the experimental evidence for specific gut bacteria and their metabolites and how they affect autism in light of the phenotypic and etiological complexity and heterogeneity. We propose a personalized medicine approach for using probiotics to increase the quality of life of individuals with autism by selecting specific probiotics to improve particular features of the condition.

## 1. Introduction

Autism spectrum disorder is the most complex and prevalent form of neurodevelopmental disorder, whose major socioeconomic burden has thus far defied any therapeutic prospect. The Centers for Disease Control and Prevention (2023) reported an increase in people on the autism spectrum from 1 in 44 to 1 in 36 children in the US. The current prevalence for adults is 1:45. The complexity of autism spectrum disorder is manifested by the extensive heterogeneity among patients, both in terms of the assortment of comorbidities and the ever-expanding spectrum of bona fide genetic and environmental causes.

The term microbiota refers to all microorganisms (archaea, eukarya (fungi, protozoa), bacteria, and viruses) that live in the body. Most of them live in the gut, where the microbiome is estimated to contain 10^14^ microorganisms [1]. The microbiota has coevolved with the host, leading mainly to symbiosis (at least one of two species benefits) and commensalism (one species benefits while the other is unaffected). However, there is increasing evidence for mutualism (a relationship in which both species benefit), as disturbances in the diversity and composition of the microbiota, known as dysbiosis, have a highly detrimental effect on host health. Microbiota dysbiosis has been repeatedly associated with autism spectrum disorder [2,3], and it has been suggested that probiotics improve the quality of life of individuals with autism spectrum disorder (the International Scientific Society for Probiotics and Prebiotics has defined probiotics as “live microorganisms that, when administered in adequate amounts, confer a health benefit on the host” [4]). There is a body of evidence that the gut microbiota interplays with the brain in a bidirectional manner via the gut–brain axis (GBA). The GBA modulates the gastrointestinal, immune, neuroendocrine, metabolic, and nervous systems.

This review first critically discusses the experimental evidence obtained from both mouse models and human studies on the role of the microbiota in the complex etiology of autism spectrum disorder and the potential of probiotics in improving the quality of life of people on the autism spectrum and then reviews the clinical trials of probiotics in autistic people to date. We included all clinical trials and case studies published in PubMed, as well as all those mentioned in previously published reviews [5,6,7,8,9,10] for which we could find available reports.

## 2. Autism Spectrum Disorder

Autism spectrum disorder is a predominantly male condition (with a 4:1 male-to-female skewed prevalence). It is characterized by impairments in social communication, repetitive and sensory behaviors, highly restricted interests, and varying levels of intellectual disability [11]. In addition to the core symptoms, people on the autism spectrum frequently have diverse co-occurring disorders like anxiety (70%), attention-deficit/hyperactivity disorder (ADHD), epilepsy, and depression [12,13,14]. Anxiety is usually manifested with phobias, including social phobias, obsessive–compulsive disorder, and generalized and separation anxiety. In addition to psychiatric and neurological disorders, gastrointestinal (GI) problems are among the most frequent comorbidities with autism spectrum disorder, which, although prevalent, are usually underestimated [14]. Dr. Leo Kanner, when describing autism in children for the first time in 1943, described “eating problems” in the majority of children presented [15]. Feeding problems, including food selectivity, such as food refusal, and poor oral intake (often related to food category and/or texture aversion), are up to five times more likely to occur in children with autism than in neurotypical children [14]. Children with autism usually prefer carbohydrates and processed food. It is noteworthy that children with autism have a higher incidence of GI problems not only compared to typical developing children but also compared to other developmental delays [16], suggesting that some changes in gut function and development may be specific to autism spectrum disorder [14]. GI problems in children with autism usually comprise constipation, diarrhea, and abdominal pain. Some studies have reported that GI problems are correlated with increased autism spectrum disorder severity [17] because GI problems are often accompanied by psychiatric problems, sleep disorders, and seizures [18,19]. Autistic children who experience abdominal pain, gas, diarrhea, and constipation tend to show increased irritability, social withdrawal, and hyperactivity compared to those without GI problems [20]. It has therefore been suggested that the challenging and unexplained behaviors of some toddlers with autism are partially due to their inability to verbalize their discomfort in response to GI problems, which they manifest in seemingly unassociated ways [21].

### 2.1. Etiology of Autism Spectrum Disorder

#### 2.1.1. Genetics of Autism Spectrum Disorder

The etiology of autism spectrum disorder is complex and still elusive. It is based on the complex interplay between genetics and environmental factors. Technological advances in next-generation sequencing (NGS), induced pluripotent stem cell (iPSC)-based technology, bioinformatics, and artificial intelligence revolutionized the field, enabling better clinical assessments, often combined with genetic testing. These improvements, on the one hand, probably partially contributed to the increase in the number of patients with autism since the diagnosis has been significantly improved, and on the other hand, they have helped us to discover an increasing number of genes potentially involved in the etiology of autism spectrum disorder and to find new potentially therapeutic avenues for the treatment of this condition.

Despite the complexity of the autism spectrum disorder etiology, twin studies have repeatedly shown a high genetic contribution to autism. As early as 1977, Folstein and Rutter convincingly demonstrated that monozygotic twins were more likely to have autism (36%, 4/11) than dizygotic twins (0%, 0/10) when they analyzed the core autism symptoms in a cohort of eleven monozygotic and ten dizygotic twins [22]. When the “broader autism phenotype” was analyzed, the numbers increased to 92% for monozygotic twins and 10% for dizygotic twins [23]. These initial results have been repeatedly reported in various twin studies and have also been confirmed in family studies, showing that the recurrence of a child with autism increases with the proportion of the genome shared with an affected sibling or parent [24]. There are currently over 1000 genes and copy number variations (CNVs) associated with autism spectrum disorder (The Simons Foundation Autism Research Initiative (SFARI) database, https://gene.sfari.org/ (accessed on 14 October 2024). The most common CNVs are 16p11.2 deletion and duplication (approximately 0.8% of all autism spectrum disorder cases), 22q11 deletion and duplication (~0.5%), 1q21.1 deletion and duplication (~0.2%), 15q11-13 duplication (~0.5%), and 7q11.23 duplication (~0.2%) (reviewed in [25]). In addition to the CNVs, mutations in many genes have been associated with autism spectrum disorder. However, the susceptibility genes converge on a limited number of biological pathways, such as transcription factors (e.g., *ADNP*, *RAI1*, *TBR1*, *FOXP2*, and *DEAF1*), chromatin remodelers (e.g., *CHD2*, *CHD7*, *CHD8*, *POGZ*, *KMT5B*, *KDM6B*, *ARID1B*, *SETD5*, and *MECP2*), protein synthesis (deregulated mRNA translation, e.g., *TSC1/2*, *PTEN*, *FMR1*, and *NF1*), protein degradation (e.g., *CUL3*, and *UBE3A*), cytoskeleton (actin) dynamics (e.g., *PTEN*, *RELN*, *TBR1*, *DLX1/2*, *AUTS2*, *NEXMIF*, *WDFY3*, and *NDE1*), and synaptic function and plasticity (e.g., *NLGNs*, *SCN2A*, *NRXNs*, *SYNGAP1*, *ANK2*, and *DLG4/PSD-95*) [24,26]. In addition to this never-ending number of genes found to be mutated in autism, it has recently been shown that common genetic variants can also account for almost all the heritability of autism spectrum disorder [27,28]. Thus, there is increasing evidence that the interaction between common and rare variants shapes the genetic landscape of autism spectrum disorder. In addition, alterations in several pathways are known to be linked with some observed comorbidities; for example, serotonin–NAS–melatonin is associated with circadian/sleep disorder, the oxytocin pathway with sociability [29], and the mitochondria with the metabolism [30]. Also, the iPSC-derived 2D and 3D neuronal models carrying CNVs or point mutations for high-confidence autism spectrum disorder genes have revealed altered kinetics of neuronal differentiation [31,32,33,34]. However, these highly penetrant mutations are associated with a more severe clinical picture, so the question is whether deregulated neuronal differentiation is also associated with the milder forms of autism spectrum disorder.

#### 2.1.2. Environmental Risk Factors for Autism Spectrum Disorder

The term “exposome” was coined by cancer biologist Christopher P. Wild in 2005 [35]. It encompasses all environmental factors to which an individual is exposed throughout life. The HUMAN EARLY LIFE Exposome (HELIX) study, a longitudinal population-based birth cohort study, identified pregnancy and the periconceptional period as developmentally critical periods during which environmental exposures can influence child health outcomes [36]. This hypothesis was further supported by the work of the CHARGE (Childhood Autism Risks from Genetics and the Environment) consortium, which identified an increased risk for autism spectrum disorder associated with maternal occupational exposure to solvents and suggested that other chemicals might have a similar negative effect on neurodevelopment [37]. Indeed, further studies in animals and humans identified several environmental risks, such as advanced parental age, extreme prematurity, very low birth weight, birth complications leading to periods of oxygen deprivation to the baby’s brain, maternal obesity, diabetes, immune system disorders, cesarean section, prenatal exposure to air pollution, certain pesticides, toxic metals, drugs, and endocrine-disrupting chemicals, as being associated with an increased risk of autism spectrum disorder. Although the underlying mechanisms are elusive, there is evidence that exposome might alter mechanisms such as oxidative stress [38], inflammation, hypoxia, neurotransmitters, and signaling pathways (reviewed in [39,40,41]). A specific combination of intrinsic host factors and exposure to drugs, toxins, pathogens, changes in diet, alterations to the immune system, and some physical and psychological conditions like stress can lead to aberrant colonization of the gut microbiota prenatally, in the first months after birth, and/or later in life, leading to dysbiosis whose consequences can be detrimental for the health of the host.

Indeed, dysbiosis has been repeatedly described in individuals with autism, although with high variability in microbes identified as deregulated (reviewed in [2]). Results can vary depending on the individual, age, and severity of autism. However, despite all differences, in most studies, a decrease in beneficial bacteria, such as *Bifidobacterium* and *Lactobacillus*, and an increase in potentially harmful bacteria, like *Clostridium* and *Bacteroides*, have been observed.

### 2.2. The Role of Microbiota in Brain Development and Autism Spectrum Disorder Risk

#### 2.2.1. Human Microbiome and Fetal Development

The gut microbiome is a dynamic ecosystem that varies between individuals and within the same individual across the lifespan. In the healthy adult gut, the phyla Firmicutes (e.g., *Enterococcus*, *Lactobacillus*, *Clostridiales*, *Bacillus*, and *Ruminicoccus*) and Bacteroidetes (e.g., *Bacteroides* and *Prevotella*) are most abundant. In contrast, the phyla Actinobacteria (*Bifidobacteria*), Fusobacteria, Proteobacteria (*Escherichia coli*), and Verrucomicrobiota are less abundant [42]. In humans, colonization of the gut microbiota begins in the womb, followed by rapid colonization immediately after birth [1].

Intrauterine brain development begins during the sixth week of gestation in humans. It follows a carefully orchestrated sequence of events regulated by specific gene expression programs. Environmental factors can influence epigenetic programming during various stages of neurodevelopment. The third trimester of human development has been identified as critical for synaptogenesis. Therefore, environmental exposures during the third trimester may disrupt synaptogenesis [43], which is a critical process implicated in autism spectrum disorder. Indeed, environmental factors, such as maternal infections, diet, metabolic status, and chemical exposures during pregnancy, have been suggested to interfere with brain development and contribute to autistic traits, highlighting the complex interplay between environmental exposures, the maternal gut microbiome, and offspring outcomes.

The maternal gut microbial community changes significantly between the first and third trimesters. Starting from the second trimester, there is an increase in Proteobacteria, *Bifidobacteria*, and lactic acid-producing bacteria (including specific *Lactobacillus* strains), accompanied by a decline in butyrate-producing bacteria. Overall, the gut microbiota during pregnancy is defined by low alpha and high beta diversity (alpha diversity represents the within-sample diversity (statistic of a single population), while beta diversity estimates similarity or dissimilarity between samples (between populations)), with the most notable changes occurring primarily in the third trimester [42], which is critical for synaptogenesis and brain formation. The described disruption of the maternal microbiota adaptation to pregnancy is called maternal gut dysbiosis. It can be caused by diet, maternal obesity, antidepressant and antibiotic use, inflammation, stress, and infection. Maternal dysbiosis affects both maternal and fetal health.

#### 2.2.2. Maternal Exposome and Autism Spectrum Disorder Risk: Maternal Immune Activation (MIA) and Maternal Obesity

In the 1970s, Stella Chess, an American psychiatrist, found that the prevalence of autism was 200 times increased compared to that of the general population in the US for children with congenital rubella syndrome after the epidemic in New York in the 1960s. Afterward, studies revealed similar findings for other viral infections (e.g., cytomegalovirus, influenza) during pregnancy [43]. Finally, a large-scale epidemiological study using the Danish Medical Register has recently confirmed and expanded upon previous research linking maternal infection to an increased risk of autism in offspring. Analysis of more than 10,000 autism cases revealed a strong association specifically with maternal viral infections occurring during the first trimester. Thus, both human and animal studies indicate that maternal inflammation, known as maternal immune activation (MIA), caused by infection and/or autoimmune disease, is a risk factor for children with autism [14]. In animals, exposure to various infections during pregnancy leads offspring to develop abnormal social behaviors, an anxiety-like phenotype, and deficits in their immune profiles (reviewed in [43]). Interestingly, it has been shown that MIA also elicits increased gut permeability and gut dysbiosis [44,45]. After treatment of MIA-exposed animals with probiotics known to elicit the host immune system and protect against *Helicobacter hepaticus*-driven colitis in mice, *Bacteroides fragilis*, they reverted to many autism-like symptoms. The treatment normalized intestinal permeability, prevented the migration of neurotransmitters through the gut wall, and improved hyperactivity, anxiety-like behavior, aberrant communication, and stereotypy, but not social dysfunction, corroborating crosstalk between microbiota and the host immune system during in-utero development in mice [44,46]. Likewise, microbiota metabolites like short-chain fatty acids (SCFAs), which can be supplemented via a high-fiber diet, have been shown to inhibit inflammation by downregulation of NF-κB and histone deacetylase (HDAC) activity [47]. Similarly, a recent in-depth study of the molecular mechanism underlying the BTBR T + Itpr3tf/J (BTBR) mouse model of idiopathic autism, which displays systemic immune dysregulation and gut dysbiosis, revealed that aberrant epigenetic regulation by HDAC1 as far as in the yolk sac is in the bases of observed phenotypes [48]. The proinflammatory phenotype was successfully rescued by administrating sodium butyrate, one of the SCFAs. Interestingly, the BTBR mouse model was originally bred to study insulin resistance, phenylketonuria, and diabetes-induced nephropathy, but since it showed strong and consistent autism-like features, it became known as a model of idiopathic autism [49], thus corroborating interplay between the immune system, microbiota, and metabolism in autism spectrum disorder and opening avenues for microbiota-based targeted treatments in autistic people.

Similarly, excessive deprivation or an abnormal increase in maternal nutrient intake, which is tightly linked to dysbiosis, impacts offspring development. For example, obesity during pregnancy is characterized by an increase in Firmicutes, which results in a high Firmicutes–Bacteroidetes ratio and is related to weight gain, gut inflammation, and increased gut permeability [50]. The correlation between maternal obesity and children with autism has been extensively studied. A large cohort study in Finland, which analyzed 649,043 births from 2004 to 2014, found that mothers with severe obesity and diabetes have a sixfold increased risk of having a child with autism and/or AHDH compared to mothers with a normal body mass index (BMI) and no diabetes [51]. Similarly, a population-based case–control study conducted in California, US, involving 1004 children aged 2–5 years between 2003 and 2010, reported that maternal metabolic conditions (diabetes, hypertension, and obesity) during pregnancy increased the likelihood of having a child with autism and developmental delay [52]. Additionally, a Swedish population-based cohort study using a family-based study design including 333,057 individuals born between 1984 and 2007, out of which 6420 had autism, was used to study the correlation between BMI, gestational weight gain, and the risk of children with autism. Although at the level of population, maternal obesity showed an increased risk of having a child with autism, in matched sibling analysis this correlation was not present, suggesting that maternal obesity could be a proxy for some other familial risk factors. Instead, they found a direct link between gestational weight gain and autism spectrum disorder [53], which could be linked to pregnancy-related maternal dysbiosis.

#### 2.2.3. The Gut Microbiome Impacts Neurodevelopment in Mice in Physiological Conditions

Although correlation studies in humans suggested the existence of GBA and the role of microbiota in brain development and function, only the experiments using germ-free animals, fecal microbiota transplantation (FMT), and antibiotic-driven changes in gut microbiome composition in the context of brain physiology and pathology proved it [54,55,56,57,58]. Sudo and colleagues demonstrated the role of microbiota in early postnatal brain development and plasticity in mice by comparing hypothalamic–pituitary–adrenal (HPA) axis response to stress in germ-free animals and specific pathogen-free mice. They found that germ-free mice were more sensitive to stress than specific pathogen-free mice, suggesting the role of microbiota in stress response [59]. Further, the authors rescued the exaggerated HPA stress response in germ-free mice by supplementation with *Bifidobacterium infantis*. The exaggerated HPA response of germ-free mice was also partially corrected early in development by FMT from specific pathogen-free mice, but the correction failed at later stages of development. Thus, the authors demonstrated that specific gut microbiota species can modulate HPA stress responses in mice during early postnatal development, corroborating the role of specific gut microbes in early brain development and its postnatal plasticity in mice.

Vuong and colleagues instead addressed the role of maternal gut microbiota in offspring brain development, function, and behavior. They showed the essential role of maternal gut microbiota during the critical period of prenatal development, and its essential role in axonogenesis [60]. Embryos from antibiotic-treated and germ-free mothers exhibited reduced expression of genes related to axonogenesis, deficient thalamocortical axons, and impaired thalamic axon outgrowth in response to cell-extrinsic factors. Colonization of antibiotic-treated mothers with Clostridia-dominant spore-forming bacteria (Sp; phyla Firmicutes) mitigated several negative impacts of maternal microbiota depletion on fetal brain gene expression and thalamocortical axonogenesis, whereas colonization with Bacteroidetes had a very mild effect or no effect. Additionally, 30 fetal brain metabolites discriminated specific pathogen-free mice and Sp versus germ-free and antibiotic-treated microbiota status. Among them, eight metabolites showed similar differential regulation also in maternal sera, indicating that maternal microbiota influences the availability of the metabolites in maternal blood, thus directly affecting metabolite availability to the fetal brains of developing offspring. The metabolites trimethylamine-N-oxide (TMAO), N, N, N-trimethyl-5-aminovalerate (TMAV), imidazole propionate (IP), 3-indoxyl sulfate (3-IS), and hippurate (HIP) were reduced more than twofold in both maternal blood and fetal brain from antibiotic-treated and germ-free mothers, compared to specific pathogen-free mice controls, and the levels were restored by maternal colonization with Sp. Exposure to murine concentrations of TMAO, 5-aminovalerate (5AV, a precursor to TMAV), IP, or HIP, but not 3-IS, significantly increased axon numbers. Furthermore, 5AV and IP promoted longer axons, whereas TMAO and 3-IS showed moderate but not statistically significant increases, and HIP had no effect. In contrast, maternal supplementation with the short-chain fatty acids propionate, butyrate, and acetate had no significant impact on fetal thalamocortical axons. Thus, this study demonstrated that mid-gestation is a crucial period when the maternal microbiome plays a key role in promoting fetal neurodevelopment, supporting developmental processes that may influence behaviors later in life, where specific bacterial strains, through their metabolites, exert distinct functions. Of note, mid-gestation in humans begins at the 13th gestation week and corresponds to an intense brain development period and dynamic maternal gut microbiota changes, thus opening the possibility for similar microbiota regulation of fetal brain development in humans as well.

#### 2.2.4. Fecal Microbiota Transplantation from Individuals with Autism into Healthy Rodent Recipients Elicits Autism-like Phenotype

Rodents are valuable tools in studying molecular mechanisms underlying autism spectrum disorder since mouse and rat behaviors can directly reflect the three core symptoms of autism: (i) social interaction deficits, assessed through tests like the three-chamber assay or video analysis; (ii) communication deficits, measured by ultrasonic vocalizations or scent marking; and (iii) increased repetitive or stereotyped motor behaviors, such as self-grooming or marble-burying. Additionally, insistence on sameness and restricted interests can be observed through neophobia or perseverative behavior in T-maze or water maze tests [61]. Mouse models, in combination with FMT, provided strong evidence for the role of gut microbiota in autism spectrum disorder. On the one hand, transplantation of gut microbiota from human donors with autism into recipient mice induced core behavioral autism spectrum disorder symptoms 3 weeks after transplantation [62] as well as in offspring [63], while on the other hand, the FMT from typically developed (TD) individuals improved valproic acid (VPA)-induced autistic features in mice [64].

Specifically, Sharon and colleagues showed that transplantation of fecal samples from individuals with autism into germ-free mice (“humanized microbiome mice”) led to behavioral deficits relevant to autism spectrum disorder, e.g., increased repetitive behavior, decreased locomotion, sociability, and communication in offspring ASD (oASD) mice compared to offspring from mice transplanted with TD control fecal samples (oTD) [63]. Donors were chosen based on the Autism Diagnostic Observation Schedule (ADOS) and GI severity index (GSI) scores and divided into three groups based on the disease severity: TD, mild ASD (standardized ADOS (StdADOS) = 4–5), or ASD (StdADOS = 6–10). Only the samples from StdADOS = 6–10 elicited behavioral deficits, which were more pronounced in males, potentially due to a bias from using microbiota from human male donors. While the authors found that some age-related microbiota could affect locomotion, increased repetitive behavior showed high correlation with ADOS and GSI scores. Importantly, no differences were detected between oTD and oASD mice for intestinal barrier function, mouse weight, or cytokines and chemokines (local inflammation) in the GI tract. Alpha- and beta-diversity changed in both recipient mice and offspring. Changes were found in Clostridia and Bacteroidia classes, with single representatives from the Verrucomicrobia, α-, and β-Proteobacteria phyla. In particular, *Bacteroides ovatus* (054dc_Bacteroides ovatus and 970ed _Bacteroides ovatus) and *Parabacteroides merdae* (4ae7e_Parabacteroides) were consistently present in oTD samples and absent from almost all oASD samples. Instead, *Eisenbergiela tayi* (29857_Lachnospiraceae and 02b40_Lachnospiraceae) was present in all oASD mice and absent in oTD groups. Additionally, β-diversity differed between oASD and oTD mice, with *Bacteroides*, Bacteroidetes, and *Parabacteroides* being decreased and *Akkermansia*, *Sutterella*, and *Lachnospiraceae* increased in oASD mice, similar to the findings in humans [63]. Of note, reduced repetitive behavior and increased social behavior correlated with *Bacteroides* spp. (b20cd_Bacteroides) and *P. merdae* (4ae7e_Parabacteroides), whereas *E. tayi* (02b40_Lachnospiraceae) had the opposite effects. These findings support the idea that certain bacteria contribute to specific symptoms of autism spectrum disorder. Transcriptomics analysis uncovered several significant, differentially expressed genes between oTD and oASD mice at P45, including the *Daglb* gene, which is involved in endocannabinoid production and impinges on axonal growth during development, similar to the findings of Vuong et al. [60]. Interestingly, 560 genes showed different alternative splicing between oASD and oTD mice, with 52 associated with autism spectrum disorder, i.e., SFARI genes, and 11 present on both SFARI and SPARK (Simons Foundation Powering Autism Research for Knowledge) gene lists, which is the list containing 157 single genes and 28 CNVs that are highly associated with autism. Most of them belong to RNA-binding proteins (RBPs), including fragile X mental retardation 1 (*Fmr1*), a gene that is required for normal cognitive development, and, when mutated, causes fragile X syndrome (FXS). In addition, out of 313 detected metabolites, 27 were significantly different in the colon contents of oASD versus oTD mice, with higher levels of amino acids, similar to those found in individuals with autism. Similar to the findings in the autism population, 5-AV (weak gamma–aminobutyric acid (GABA)_A_ receptor agonist) was significantly lower in oASD mice (the same as in Vuong et al. [60]), as were the levels of taurine, another weak GABA_A_ agonist (and a potent glycine receptor agonist). Data integration by using HUMAnN2 [65] for metagenomic data and MIMOSA [66], which correlates bacterial species and their genes with the production and degradation of metabolites measured by nuclear magnetic resonance (NMR) and gas chromatography–mass spectrometry (GC-MS) analysis, uncovered that the *Akkermansia*, *Alistipes*, and *Bacteroides* genera specifically affected the metabolism of various amino acids (e.g., proline, taurine, glutamate, and glutamine). Treatments of the BTBR mouse model (see above) during the prenatal period with either taurine or 5AV led to a significant reduction in repetitive behavior and increased social behavior. Thus, this outstanding study convincingly showed that autism-specific human microbiota can promote autism-like behaviors in wild-type mice. However, it is important to highlight that this study did not conclude that gut bacteria are the sole cause of the autism spectrum disorder phenotype. It has been proven, without any doubt, that host genetics and other perinatal events affect autism spectrum disorder phenotype. Thus, this study showed that changes in microbiota could also contribute to the etiology of autism spectrum disorder by increasing the risks and/or enhancing the symptom severity. Interestingly, and unexpectedly, Sharon and colleagues showed that microbiota, besides altering the amino acid metabolism, affect alternative splicing, including some important autism-related genes, such as *Fmr1*. Likewise, it was recently shown that FMRP regulates alternative splicing and that in *Fmr1* knockout (KO) mice, alternative splicing is deregulated, affecting genes associated with autism spectrum disorder [67], similar to the findings of Sharon and colleagues. Moreover, alternative splicing has already been described in autism patients as a transcriptome-wide phenomenon [68] and is getting increasing attention lately as a possible autism biomarker [69,70]. Therefore, the fact that changes in alternative splicing were triggered by fecal samples transplanted from autism individuals into healthy mice suggests that microbiota dysbiosis could also play a role in deregulated alternative splicing observed in humans. Identification of the specific microbes underlying the deregulation of alternative splicing could lead to potential therapeutics or early diagnosis and prevention by controlling and eventually targeting gut microbiota during pregnancy.

#### 2.2.5. Fecal Microbiota Transplantation from Typically Developed Individuals Rescues Autism-Related Phenotype in Animal Models

VPA, an HDAC inhibitor, is a branched short-chain fatty acid derived from naturally occurring valeric acid. It is primarily used as a drug for epilepsy and seizures but also for migraine, anxiety, bipolar mood, and psychiatric disorders [71]. However, several studies reported that exposure to VPA during pregnancy increased the risk of autism in children [72,73]. Consequently, the Food and Drug Administration (FDA) recommended that pregnant women not take VPA and derivates to prevent migraine headaches, while for epilepsy and bipolar disorder, VPA derivates can be prescribed only if other medications are not effective or cannot be used for another reason. Similarly, rodents prenatally exposed to VPA also show autistic-like features (increased stereotypic and repetitive behaviors, reduced social interactions, changes in sensory sensitivity, impaired prepulse inhibition (PPI), increased anxiety, deficits in reversal learning, modified eye-blink conditioning, and intensified fear memory processing), making it a valuable model to study the molecular mechanisms underlying observed autism-like features [64]. Thus, Wang and colleagues used this model to assess the role of gut microbiota. They treated the VPA mice with the antibiotic for a week and then subjected them to the FMT from autism and TD donors or vehicle solution. Animals were treated every other day for 21 days and then subjected to behavioral tests. VPA mice that received FMT with TD samples had restored recognition behavior and social novelty responses, were less anxious and more likely to explore the unknown area, and had higher locomotion. Animals receiving FMT with samples from autism donors had aggravated anxiety. Altogether, these results suggest that microbiota impact core autism symptoms in mice. The microbiota analysis revealed that mice receiving FMT from autism donors had higher relative abundances of the genera *Bacteroides* and *Odoribacter.* On the other hand, the TD FMT-treated group had an increase in the genera *Akkermansia* and *Erysipelatoclostridium*, in particular, *Turicibacter* and *Alistipes.* Untargeted metabolomic profiling revealed alterations in glutathione, L-glutamic acid/(S)-glutamic acid, L-asparagine, oxidized L-proline, and thromboxane B2 (TXB2), which are involved in glutamatergic synapse metabolism and GABAergic synapse pathways. Integrating metabolomics and transcriptomics data indicated changes in glutamatergic synapse and serotonergic synapse pathways. Interestingly, high levels of free amino acids (in particular, Glu, Ala, Asp, and Lys) have already been detected in children with autism [74]. The increased level of free amino acids was correlated with the abundance of proteolytic genera (e.g., *Clostridium* and *Bacteroides*). Similarly, in another studies, GABA was found to be 36% lower in the feces of individuals with autism compared to TD [75]. These metabolomics and VPA mice data fit well with the imbalance between excessive glutamate and deficient GABA neurotransmission, described as one of the important underlying mechanisms of autism. GABA is the main inhibitory neurotransmitter in the mammalian brain. It exists naturally in various foods and can be obtained from a normal diet [76]. There are also many food supplements with GABA that are freely available in the EU and US markets. However, there has been, and still is, a thorough debate on whether GABA can pass the blood–brain barrier (BBB). While some researchers think that a very low amount of GABA can pass the BBB, some think that it can pass the BBB through various mechanisms, such as transcytosis, carrier-mediated transport, or simple diffusion of hydrophobic substances, and others that GABA acts via the enteric nervous system (reviewed in [76,77]). Nonetheless, to date, no data confirm GABA’s BBB permeability in humans. Yet, gut microbiota produce GABA—in particular, lactic acid bacteria and bifidobacteria (LAB; [77,78]). The *Bifidobacteriaceae* family, together with *Streptococcaceae*, is associated with a higher abundance of fecal GABA in healthy individuals [77,79]. In addition to *Bifidobacterium*, *Bacteroides*, and *Lactobacillus*, it has recently been shown that *Lactococcus*, a genus of lactic acid bacteria, can produce GABA as well [77]. Interestingly, in almost all neurological and psychiatric disorders (autism, schizophrenia, Parkinson’s disease, Alzheimer’s disease, epilepsy, anxiety, depression, stress, etc.), dysbiosis affects GABA producers, and it is reflected in GABA levels in patients (reviewed in [77]), putting in focus how the gut microbiota and GABA metabolism play a role in brain physiology and pathophysiology. Thus, many researchers, including our team, are actively studying the potential of GABA-producing strains as novel probiotics for co-treatment or a food supplement for different neurological and psychiatric disorders (the Science Fund of the Republic of Serbia, the IDEAS program, Project No. #7744507, NextGenBiotics). In the autism spectrum disorder field, several preclinical studies and pilot clinical trials with GABA agonists have been performed. For example, acamprosate was used in a 10-week open-label trial involving 12 subjects with FXS and comorbid autism spectrum disorder, where 75% of participants showed significant improvements, particularly in social behaviors, hyperactivity, and communication skills, as measured by the Aberrant Behavior Checklist (ABC) Social Withdrawal and Avoidance subscales [80], and STX209, a GABA_B_ agonist, reversed the autism-like behavior in the VPA-mice model [81]. Thus, it is promising to see the effect of GABA-producing probiotics on autism spectrum disorder phenotype.

## 3. Microbiota-Based Interventions for Autism Spectrum Disorder

### 3.1. Treatment with Limosilactobacillus (L.) reuteri Rescues Autism-Associated Impaired Social Behavior

Oxytocin is a neuroendocrine hormone produced in the hypothalamus that regulates social behavior [82] and colonic hyperpermeability via the vagus cholinergic pathway in rats [83]. Thus, targeting oxytocin, in addition to impacting social behavior directly, could also mitigate GI symptoms and GI symptom-related maladaptive behavior such as social withdrawal, irritability, interfering repetitive behavior, and hyperactivity [20]. However, clinical trials with intranasal administration of oxytocin did not yield consistent results, probably because intranasal oxytocin does not cross the BBB efficiently and thus may not mimic the endogenous oxytocin pattern and stimulation of the oxytocin receptor in the CNS correctly. Therefore, the fact that *Limosilactobacillus* (L.) *reuteri* (formerly known as *Lactobacillus reuteri*) has been shown to stimulate oxytocin production [84] elicited a lot of attention among scientists, especially because *L. reuteri* is declared to be ‘‘generally recognized as safe’’ (GRAS) for use in humans by the United States FDA. Here, we will describe efforts from two independent teams to assess the effect of *L. reuteri* on individuals with autism.

Buffington and colleagues found that a maternal high-fat diet (MHFD) induced changes in gut microbiota dysbiosis in offspring, characterized by a reduction in phyla Firmicutes, species *L. reuteri*, which was accompanied by a reduction in sociability and synaptic plasticity in the ventral tegmental area and fewer oxytocin neurons in the hypothalamus [85]. The authors managed to rescue social deficits by treating offspring with *L. reuteri*. To follow up on that finding, they further performed a double-blind randomized placebo-control clinical trial with *L. reuteri* (a product containing a combination of strains of ATCC-PTA-6475 and DSM-17938) on a cohort of 43 subjects, where 21 were treated with an *L. reuteri* combination and 22 were treated with a placebo (the details of the study are shown in Table 1 and Table 2), which confirmed an improvement in social deficit in humans as well, and showed an increase in adaptive social functioning, whereas no impact on overall autism spectrum disorder severity was detected [86]. Interestingly, the authors found that ATCC PTA 55730 (the parental strain of the DSM 17938) failed to reverse social deficits in an autism mouse model, suggesting a strain-specific effect, which points to the uniqueness of the metabolome of each *L. reuteri* strain.

The study by L. Schmitt and colleagues further supported using *L. reuteri* in children with autism [87]. It was given as the SB-121 formulation, consisting of *L. reuteri* (ATCC 23272), Sephadex^®^ (dextran microparticles), and maltose, which should increase adherence to intestinal epithelial cells, improve gastric survival, and enhance persistence through biofilm formation [87]. The cohort was composed of 15 males with autism with variable cognitive skills and severity of core symptoms. The treatment was safe and well tolerated, and improved adaptive behavior in all tested subjects. Some participants responded better than others, indicating subpopulations of patients that are better responders and the need for patient stratification.

The similar outcomes of two independent clinical trials corroborated the positive effect of *L. reuteri* on social functioning in individuals with autism. As the authors of both studies suggested, to further improve these positive results, the next steps should include the characterization of responders versus non-responders (clinically, but also the starting levels of oxytocin and microbiota profiles), more female participants, and clinical trials with larger sample sizes. Last but not least, it would be important to understand the difference in metabolomic and peptidomic signatures between effective *L. reuteri* ATCC PTA 6475 and ATCC 23272 strains vs. non-effective ATCC PTA 55730 strains.

**Table 1:** Clinical studies with probiotics for individuals with autism.

Study No.	Probiotic	Duration	Subjects (Age Range)	Autism Scale	GI Symptoms (Added and Exluded)	Reference
1.	DSF	6 months	46 children with autism; probiotic:placebo = 1:1; M = 35, F = 11; age: 18–72 months	ADOS, CSS	Yes	Billeci et al., 2023 [88]
2.	Probiotic contained 6 strains of bacteria, each with 1 billion CFUs/gram.	4 weeks	65 children with autism, out of which 37 received a probiotic and 28 were in the placebo group; M:F = 5:1; control group: 40 TD children; M:F = 1:1; age: 3–8 years	ATEC	Yes, 22 out of 37	Niu M et al., 2019 [89]
3.	*Bifidobacterium* sp.; *Lactobacillus* sp.; 10^8^ bacteria/g, 10 g/day	3 months	40 children with autism; age: 2–5 years	CARS	Yes	Meguid et al., 2022 [90]
4.	*Bifidobacterium* Triple Live Bacteria Powder, 2.0 × 10^7^ CFUs	3 months	41 children with autism were enrolled, 21 in the observation group and 20 in the control group; M = 16, F = 5; age: 3–6 years	ATEC, ABA	N/A	Li YQ et al., 2021 [91] (only abstract available in English)
5.	*Limosilactobacillus fermentum* LF10; *Ligilactobacillus salivarius* LS03; *Lactiplantibacillus plantarum* LP01; mix of five strains *B. longum* DLBL(07-011); 10 × 10^9^ CFUs/AFU/day	3 months	61 individuals with autism, of which 31 received the probiotic and 30 the placebo; age: 2–16 years	PSI, VABS, PEP-3, ASRS	Yes; GSI; excluded: celiac disease, Crohn’s disease, and ulcerative colitis	Guidetti et al., 2022 [92]
6.	*Lb. plantarum* PS128 3 × 10^7^ CFUs/day	4 weeks	71 boys with autism, of which 36 received the probiotic and 35 the placebo; age: 7–15 years	CGI-I, ABC-T, SRS, SNAP-IV	N/A	Liu YW et al., 2019 [93]
7.	*Lb. plantarum* PS128; oxytocin; 6 × 10^10^ CFUs/capsule, 2 capsules/day	28 weeks	Stage 1: individuals with autism, of which 18 received the probiotic and 17 the placebo; age: 3–20 years; stage 2: all participants received intranasal oxytocin spray	SRS, ABC, CGI	Yes; GSI	Kong et al., 2021 [94]
8.	*B. infantis* Bi-26, *Lb. rhamnosus* HN001, *B. lactis* BL-04, and *Lb. paracasei* LPC-37, 10^10^ CFUs/pack day	(30, 60, 108 days)	26 children with autism, out of which 16 received the probiotic and 10 the placebo; M = 24, F = 2 and 24 placebo; M = 22, F = 2; age: 2–8 years; 7 children completed the treatment, and 8 out of 10 completed the placebo supplement for 30 days	ATEC	Yes; modified version of the 6-GSI	Wang Y et al., 2020 [95]
9.	DSF 450 billion probiotic strains/packet; first month: 2 packets/day; following 5 months: 1 packet/day	6 months	63 children with autism (31 with probiotic, 9 with GI, 22 without GI symptoms) and 32 with the placebo (8 with GI, 24 without GI symptoms); age: 18–72 months	DSM-5; ADOS-2	Yes; total GSI, total 6-GSI excluded: celiac disease, gastroesophageal reflux, food allergies, gluten-free diet, casein-free diet, high-protein diet, ketogenic diet	Santocchi et al., 2020 [96]
10.	DSF (VSL#3) 900 billion bacteria/packet; first 4 weeks: ½ packet twice daily, last 4 weeks: 1 packet twice daily	8 weeks probiotic/placebo, 3-week washout, then 8-week crossover	10 female children with autism (6 with probiotic/placebo and 4 with placebo/probiotic); age: 3–12 years	DSM-5 anxiety, and GI symptoms	Yes; at least one scale of the GI module of the PedsQL scale; excluded: IBD, celiac disease, eosinophilic and esophagitis	Arnold et al., 2019 [97]
11.	*Lb. plantarum* WCSF1	12 weeks	Children with autism; age: 3–16 years	DBC	Yes	Parracho et al., 2010 [98]
12.	BCP + *B. longum* subsp. *infantis* (UCD272)	5 weeks	8 children with autism; age: 2–11 years	ABC, GIH	Yes; GI co-morbidities (including IBS, chronic constipation, diarrhea)	Sanctuary MR et al., 2019 [99]
13.	*Lb. acidophilus; Lb. rhamnosus; B. longum.* 10^8^ CFUs/day	3 months	Children with autism; age: 5–9 years	ATEC	Yes; modified 6-GSI	Shaaban et al., 2018 [100]
14.	Any type of probiotic	ND	57 children with autism (19 with a probiotic and 38 with the placebo) and 39 TD; age: 2.5–18 years	ATEC, PDD/NOS, Asperger’s	Yes; 6-GSI	Adams et al., 2011 [101]
15.	Lacetol Fort: *Lb. delbruekii* and *Lb. fermentum*, 2 × 10^8^ CFUs/day	12 weeks	100 children with autism (50 with the probiotic, 50 controls); age: 2–10 years	ATEC	Yes; 6-GSI	EI-Alfy, M.S.E. et al., 2019 [102]
16.	*Lb. acidophilus* Rosell-11 5 × 10^9^ CFUs per gram twice a day	2 months	Children with autism; age: 4–10 years	N/A	Yes; GI problems, including abdominal pain, constipation, and diarrhea	Kałużna-Czaplińska and Błaszczyk 2012 [103]
17.	Delpro^®^, 2 billion CFUs per strain, and 8 mg of Del-Immune V^®^ powder	6 months	32 children with autism; age: 3–16 years	ATEC	Yes; GI distress	West, R. et al., 2013 [104]
18.	“Children Dophilus”: 3 *Lactobacillus* strains, 2 *Bifidobacterium* strains, 1 *Streptococcus* strain (60:25:15 ratio) (exact strains were not reported)	4 months	10 children with autism, 9 non-autistic siblings, and 10 TD; age: 2–9 years	ADI	Yes; strong positive correlation between autism severity and the severity of GI dysfunction	Tomova, A. 2015 [105]
19.	*Lb. rhamnosus*, *Lb. paracasei* and *B. longum*	6 weeks	Children with autism; age: 9–12 years	ATEC	N/A	Tharawadeephimuk et al., 2019; only abstract available [106]
20.	*Lb. plantarum* PS128 (3 × 10^10^ CFUs) 105 patients with *Lb. plantarum* PS128, 26 with other probiotics (OP group)	3 months or longer	131 children with autism, *Lb. plantarum*: 105 subjects, M:F = 122:19; age: 86.1 ± 41.1 months; 26: OP group; age: 45–127 months	ADOS-2, CGI (CGI-severity of 5 or higher: 113, 86.3%)	Yes; 52 had GI problems	Mensi et al., 2021 [107]
21.	SB-121	28 days (probiotic/placebo); after a 14-day washout, switched to the other treatment (probiotic/placebo) for 28 days	15 participants with autism 15–45 years old (7 probiotic/placebo and 8 placebo/probiotic)	ADOS-2	No	Schmitt et al., 2023 [87]
22.	*Lb. reuteri* DSM 17938, *Lb. reuteri* ATCC PTA 6475	6 months	43 children (probiotic: 21 participants, M = 19, F = 2; age 5.8 ± 1.3 years; placebo: 22 participants, M = 16, F = 6; Age 5.5 ± 1.2 years)	ADOS-2	Yes; not observed improvement in GI symptomatology	Mazzone L, et al., 2024 [86]
23.	DSF (VSL#3)	4 weeks and 4-month follow-up	Case report of a boy with autism, 12 years old	Severe cognitive disability, DSM-V ADOS-2	Yes	Grossi et al., 2016 [108]
24.	*Saccharomyces boulardii* (Klaire Labs, 3 billion CFUs per capsule) initially: 6 capsules/day, a final dose: 12 capsules/day	ND	Case report of a 16-year-old boy	Severe OCD, tics, SIB	Yes; the unique benefits of promoting normalization of gut flora, improving immunomodulation, and managing recurrent diarrhea	Kobliner et al., 2018 [109]

Males—M; females—F; typically developed—TD; gastrointestinal—GI; GI Severity Index—GSI; Diagnostic and Statistical Manual of Mental Disorders-5th Edition—DSM-5; Autism Diagnostic Observation Schedule, Second Edition—ADOS-2; applied behavior analysis—ABA; inflammatory bowel disease—IBD; Pediatric Quality of Life Inventory—PedsQL; obsessive compulsive disorder—OCD; severe self-injurious behavior—SIB; Psychoeducational Profile, Third Edition—PEP-3; formulations of mixed probiotics: De Simone Formulation—DSF (Vivomixx^®^ in the EU and Visbiome^®^ (VSL#3) in the US): *St. thermophilus* NCIMB 30438, *B. breve* NCIMB 30441, *B. longum* (reclassified as *B. lactis*) NCIMB 30435, *B. infantis* (reclassified as *B. lactis*) NCIMB 30436, *Lb. acidophilus* NCIMB 30442, Lb. *plantarum* NCIMB 30437, *Lb. paracasei* NCIMB 30439, and *Lb. delbrueckii* subsp. *bulgaricus* (reclassified as *L. helveticus*) NCIMB 30440; *Bifidobacterium* Triple Live Bacteria Powder: *B. longum*, *Lb. acidophilus*, and *E. faecalis*; Delpro^®^: *Lb. acidophilus*, *Lb. casei*, *Lb. delbruecki*, *B. longum*, *B. bifidum*, and 8 mg of Del-Immune V^®^ powder (a lysed, lyophilized powder that contains peptidoglycan, muramyl peptides, and nucleotide-containing components or DNA motifs that is derived from *L. rhamnosus* V strain); SB-121: a formulation of *L. reuteri* (ATCC 23272) with Sephadex and maltose.

**Table 2:** Effect of probiotics on the health of individuals with autism.

Study No.	Changes in Microbiota	Effect of Probiotic Administration	Reference
1	NA	Decreased frontopolar power and increased frontopolar coherence in beta and gamma bands in EEG.	Billeci et al., 2023 [88]
2	Phylum Bacteroidetes is lower in the autism group, while Firmicutes is higher. The genera *Bacteroides*, *Bifidobacterium*, *Ruminococcus*, *Roseburia*, and *Blautia* are lower, while *Lachnospira* is higher in the autism group.	Decreased ATEC total scores; improvements in speech/language communication, sociability, sensory/cognitive awareness, GI symptoms, and health/physical/behavior (the greatest decline)	Niu M et al., 2019 [89]
3	Increase in *Bifidobacterium* sp. and *Lactobacillus* sp.	Decreased weight, BMI, hyperactivity, and GI symptoms; improved CARS, sleep disturbances, communication, and social networking	Meguid et al., 2022 [90]
4	Higher relative abundances of *Bifidobacterium*, *Lactobacillus*, *Coprobacillus*, *Ruminococcus*, *Prevotella*, and *Blautia*, and lower relative abundances of *Shigella* and *Clostridium*	Improved clinical symptoms and corrected dysbiosis	Li YQ et al., 2021 [91] (only abstract available in English)
5	Presence of some taxa coherent with the probiotic formulation *Bifidobacterium longum*, *Limosilactobacillus fermentum*, and *Ligilactobacillus salivarius* species, while *Bifidobacterium longum* species were found after placebo supplementation, consistent with the bifidogenic effect of maltodextrins	Improved GI symptoms, communication skills, and maladaptive behaviors; reduced parental stress levels	Guidetti et al., 2022 [92]
6	N/A	Meliorates disruptive behaviors, hyperactivity/impulsivity; more effective in younger children, underscoring the importance of early intervention	Liu YW et al., 2019 [93]
7	Probiotic + oxytocin group:: *Christensenellaceae* R7, *Ruminococcaceae* UCG-002, *Lachnospiraceae* UCG-001, *Blautia*, and *Barnesiella*; Oxytocin e group: *Coprococcus*-2, *Rikenellaceae* RC9, *Bilophila*, *Catenibacterium*, and *Holdemanella*; Both probiotic groups: *Roseburia*, *Veillonella*, and *Streptococcus. Eubacterium hallii* was negatively correlated with social behavior scores.	Improved autism scale (ABC and SRS) and GI symptoms	Kong et al., 2021 [94]
8	Treatment group: gradual decrease in alpha diversity at 30, 60, and 108 days; beta diversity significantly distinct from baseline (0 days) to 108 days. Significant increase in *Bifidobacteriales* and particularly *Bifidobacterium longum* and a notable reduction in the abundance of *Clostridium and Ruminococcus*.	Treatment group: after 30 days of treatment, decrease in overall ATEC scores, but it was not statistically significant From 30 to 60 days, the overall ATEC scores significantly decreased. At 108 days, ATEC scores showed significant improvement compared to the 60-day point. Significant increase in the three SCFAs and increasing trend in the concentrations of isobutyric acid and caproic acid in the feces of the treated group.	Wang Y et al., 2020 [95]
9	N/A	Improved GI symptoms, adaptive functioning, and sensory profiles in children with autism and GI symptoms; improvement in ADOSCSS in children with autism without GI symptoms	Santocchi et al., 2020 [96]
10	No treatment-associated shifts in microbiota α-diversity or family-level composition were observed. *Lactobacillus* OTU GM884480.1.1531 was significantly correlated with the PedsQL score.	Safe, with suggested health benefits for GI symptoms in children with autism; efficacy for quality of life unproven	Arnold et al., 2019 [97]
11	Significantly increased Lactobacilli and Enterococci and significantly reduced counts of Clostridium cluster XIVa compared to placebo.	Probiotic treatment also improved stool consistency and behavior scores compared to baseline.	Parracho et al., 2010 [98]
12	No changes were observed in bacterial taxa like Clostridia and Desulfovibrio that differ between children with autism and typically developing children.	Increased appetite and willingness to consume novel foods; significant changes in intracellular cytokine expression and a reduction in helper T lymphocytes expressing IL-13	Sanctuary MR et al., 2019 [99]
13	Increase in *Bifidobacteria* and *Lactobacilli*	Decreased body weight; improvement in ATEC and GI symptoms	Shaaban et al., 2018 [100]
14	Decrease in *Bifidobacteria* species and 100% higher levels of *Lactobacillus* species (*p* = 0.00002) than in TD children.	Decrease in total and individual SCFAs in the probiotic ADS group compared to placebo. Lysozymes were lower in the autism group compared to controls, and the lower levels of lyszoymes were associated with probiotic usage.	Adams et al., 2011 [101]
15	N/A	Reduction in GSI-6	EI-Alfy, M.S.E. et al., 2019 [102]
16	N/A	Decreased D-arabinitol and D-arabinitol/L-arabinitol ratio in urine; significant improvement in concentration and following orders, with mixed results for reactions to emotions or eye contact	Kałużna-Czaplińska and Błaszczyk 2012 [103]
17	N/A	Decreased ATEC score with improvements in speech, language communication, sociability, sensory cognitive awareness, health/physical behavior, and GI symptoms	West, R. et al., 2013 [104]
18	After probiotic supplementation in children with autism, the Bacteroidetes/Firmicutes ratio was normalized, and the amount of *Bifidobacterium* and *Desulfovibrio* decreased significantly.	N/A	Tomova, A. 2015 [105]
19	N/A	Improvement in ATEC, including in speech/language communication, sociability, sensory/cognitive awareness, and health/physical behavior	Tharawadeephimuk et al. 2019; only abstract available [106]
20	N/A	Improved CGI scores, with younger age correlating with more positive probiotic effects	Mensi et al., 2021 [107]
21	N/A	Improved Vineland-3 scores compared to placebo in all tested subjects; trends toward improved social preference	Schmitt et al., 2023 [87]
22	No alteration in microbiome composition or immune profile was observed.	No alteration in overall autism severity or restricted/repetitive behaviors, but significant improvements in social functioning	Mazzone L, et al., 2024 [86]
23	N/A	Improvements in the social affect domain of ADOS and abdominal symptoms	Grossi et al., 2016 [108]
24	N/A	Reduced obsessive-compulsive disorder and self-injurious behavior	Kobliner et al., 2018 [109]

### 3.2. Clinical Trials with FMT for Autism Spectrum Disorder: Pros and Cons

FMT has been successfully used to treat recurrent *Clostridium difficile* infection [110] and was suggested as the treatment of choice for various GI problems. The first open-label modified FMT trial to treat children with autism and chronic GI problems was performed by Kang and colleagues [111]. They used an intensive microbial transfer therapy (MTT) consisting of two weeks of vancomycin treatment to deplete endogenous gut bacteria, followed by a bowel cleanse and then high-dose FMT for 1–2 days and 7–8 weeks of daily maintenance of doses along with a stomach-acid suppressant. The treatment lasted for 18 weeks in total (10 weeks of treatment plus 8 weeks of follow-up), and the authors observed an 80% reduction in GI symptoms and a slow but steady improvement in core autism symptoms. They found a significant increase in the abundance of *Bifidobacterium*, *Provotella*, and *Desulfovibrio*. While *Bifidobacterium* and *Provotella* were known to be depleted in children with autism, the increase in *Desulfovibrio* was unexpected, as it is described as both commensal and detrimental [111]. In the follow-up, two years after the treatment was completed, most improvements in GI symptoms were still maintained, and autism-related symptoms improved even more after the end of the treatment [112]. However, although FMT is generally considered safe, concerns have arisen about the risk of infection transmission after the FDA reported two cases of extended-spectrum beta-lactamase (ESBL)-producing *Escherichia coli* (*E. coli*) infection (one death) and six cases of Shiga toxin-producing *E. coli* infection (two deaths), probably related to the donor stool [113]. It is estimated that 4% of recipients develop new infectious diseases between 1 and 6 months after transplantation [113]. In addition, FMT safety has not been assessed in high-risk patients, like chronic immune-mediated conditions, where exposure of patients to allergenic strains from FMT could be detrimental. Moreover, the success of the FMT strongly depends on the composition of the donor’s stool microbiome profile [114]. It is worth mentioning that fecal samples, besides bacteria, contain viruses, fungi, and small molecules, which may influence the altered gut microbiome of the recipient. Last but not least, there is still an open question of maintaining the colonized microbiome in the recipients’ gut environment.

### 3.3. Clinical Trials with Probiotics 

While there are still too many unanswered questions related to the success of the FMT, probiotics represent a safe and reliable tool for microbiota modulation. Most of the probiotic strains belong to species that have a long history of safe use in foods or belong to commensal gut bacteria. However, there are certain criteria that each probiotic strain should fulfill to be approved for use in clinical trials, or generally for human use, out of which the most important are susceptibility to antimicrobials and the absence of virulent features (e.g., hemolytic capacity). Thus, bearing in mind that there is no effective treatment for core autism spectrum disorder symptoms, the safety of probiotics, and a wealth of promising preclinical data, including FMT data in both animal models and humans, many pilot clinical trials have been performed to assess their potential effect on the health of individuals with autism (Table 1 and Table 2).

However, despite promising preclinical studies and FMT data, clinical trials performed so far with probiotics have only been in the form of pilot studies with a limited number of participants and single-center-based. We found available data for 23 clinical trials and 2 case studies with only one participant, among which 4 studies had the same combination of probiotics—DSF. Yet, it is difficult to compare the outcomes because of the lack of consistency between studies. In particular, the clinical studies significantly differ in terms of (i) the age of participants—it is known that the gut microbiota changes dynamically during childhood, so it is difficult to compare the effect of probiotics in toddlers, schoolchildren, teenagers, and adults. (ii) Differences in probiotics used and protocols applied—it would be highly recommended to use the same combinations of probiotics, applied with the same standardized protocol in at least two independent centers, or perform multi-centric studies to generate more reliable findings. (iii) Differences in measured outcomes, and in questionnaires and surveys used to measure the outcomes—it would be important to always use the same standardized and widely accepted questionnaires and tests to measure the outcomes. Using self-reported questionnaires and surveys for measuring responses in open-label trials is prone to bias. As already discussed, due to the communication problems that are common in autism conditions, it might be challenging for parents or caregivers to answer some questionnaires. (iv) Differences in inclusion and exclusion criteria. 

Out of these 25 studies, only 2 studies with *L. reuteri* [86,87] are comparable, and indeed, they gave reproducible results, as discussed above. The authors of both studies found that *L. reuteri* does not affect autism severity or restricted/repetitive behaviors but does significantly improve social functioning. They also suggested performing in-depth analyses of both responders and non-responders to identify potential biomarkers for patient stratification. Indeed, this strategy has already been proven successful in the case of *Bifidobacterium longum* BB536, known to regulate bowel function, in a randomized, double-blind, controlled crossover trial for patients with constipation. Enrolled subjects showed great variability in responses, and after analyzing the metabolome and microbiome of both responders and non-responders, it was identified that the level of butyrate produced and the location of microbes in the colon were good markers of whether a subject would respond to *B. longum* BB536 or not. We believe that this approach could also be useful in the autism spectrum disorder field. In addition, the field would highly benefit from more randomized and controlled studies with more participants. Altogether, despite encouraging preclinical data, the overall outcome from all clinical trials with probiotics is rather disappointing because there is no possibility of building a consensus about their efficacy in individuals with autism. 

## 4. Conclusions

In this review, we have provided a number of examples of the heterogeneity of autism spectrum disorders, both in terms of etiology and phenotypes of individuals with this condition. The complexity underlying the autism spectrum disorder-specific microbiota was discussed in a recent review [2]. Here, we highlighted only bacteria associated with specific phenotypic characteristics (metabolites, molecular mechanism, clinical features) that could be used to stratify patients in further clinical trials. The inconsistencies in the clinical studies conducted so far suggest that the effects of probiotics on autism spectrum disorders cannot be generalized, and indicates the need for a personalized and precise approach in order to target clearly defined symptoms of autism spectrum disorder. In addition to standardized protocols for probiotic treatments, it would be important to select specific primary and secondary outcomes along with probiotics that have already been experimentally shown to be associated with the selected phenotype (e.g., butyrate producers for deregulated immune response). Given the complexity of autism spectrum disorder, it is difficult to imagine that “one probiotic fits all” will produce the desired results. Thus, based on the animal research and human population studies discussed here, it would be interesting to stratify individuals with autism based on their immunological and metabolomic profiles, which are widely accessible via medical examinations, and then select groups for treatments with selected probiotics. More advanced patient stratification would include integration of genetics analysis (whole exome/genome sequencing, WES/WGS), microbiota analysis (metagenomics), and probably HDAC activity, which in the era of machine learning and big data integration is probably not far away. A personalized approach will likely be necessary to reduce outcome complexity and stratify patients, which, together with scientific rigor in protocol application and treatment analysis, will more likely lead to the expected outcome.

### Study Limitations

There are several limitations to this study. Some, such as a lack of basic information like, for example, which probiotics were used in each of the 25 clinical trials, are the fault of the study authors. Others stem from incomplete knowledge about, on the one hand, probiotics—we still do not know which metabolites are produced by each of the bacteria used in the clinical trials—and, on the other hand, people with autism in terms of their microbiota, metabolite, and immune profiles, as well as genetics. This makes it difficult to properly stratify patients and select the right probiotics. All these limitations make it difficult to compare studies and objectively assess the effect of probiotics to date.

## Abbreviations

3-IS	3-Indoxyl sulfate
5AV	5-Aminovaleric acid
ABA	Applied behavior analysis
ABAS-2	Adaptive Behavior Assessment System, Second Edition
ABC	Aberrant Behavior Checklist
ABC-T	Aberrant Behavior Checklist-T
ADHD	Attention-deficit/hyperactivity disorder
ADOS	Autism Diagnostic Observation Schedule
Ala	Alanine
ASD	Autism spectrum disorder
Asp	Aspartic acid
ASRS	Autism Spectrum Rating Scale
ATCC	American Type Culture Collection
ATEC	Autism Treatment Evaluation Checklist
BBB	Blood–brain barrier
BDNF	Brain-derived neurotrophic factor
BMI	Body mass index
BTBR	Black and Tan BRachyury
CARS	Childhood Autism Rating Scale
CFU	Colony-forming units
CGI-I	Clinical Global Impression-Improvement
CHARGE	Childhood Autism Risks from Genetics and the Environment
CNS	Central nervous system
CNVs	Copy number variations
CSS	Calibrated Severity Score
DBC	Developmental Behavior Checklist
DM	Dextran microparticles
DSM-5	Diagnostic and Statistical Manual of Mental Disorders-5th Edition
EEG	Electroencephalography
EU	European Union
FDA	Food and Drug Administration
FMT	Fecal microbiota transplantation
FOS	Fructooligosaccharides
FXS	Fragile X syndrome
Fmr1	Fragile X mental retardation 1
FMRP	Fragile X mental retardation protein
GABA	Gamma–aminobutyric acid
GBA	Gut–brain axis
GC-MS	Gas chromatography–mass spectrometry
GI	Gastrointestinal
GIH	Gastrointestinal health
Glu	Glutamic acid
GRAS	Generally recognized as safe
GSI	GI Severity Index
HDAC	Histone deacetylase
HELIX	The HUMAN EARLY LIFE Exposome
HIP	Hippurate
HPA	Hypothalamic–pituitary–adrenal
IBS	Irritable bowel syndrome
IL-13	Interleukin 13
IP	Imidazole propionate
iPSC	Induced pluripotent stem cells
KO	Knockout
LAB	Lactic acid bacteria
Lys	Lysine
MIA	Maternal immune activation
MTT	Microbial transfer therapy
NAS	N-acetylserotonin
NGS	Next-generation sequencing
NMR	Nuclear magnetic resonance
NOS	Not otherwise specified
oASD	Offspring ASD
oTD	Offspring typically developed
PDD	Pervasive developmental disorder
PedsQL	Pediatric Quality of Life Inventory
PEP	Psycho-Educational Profile
PSI	Parenting Stress Index
rASD	Recipient ASD
RBPs	RNA-binding proteins
RNA-seq	RNA sequencing
rTD	Recipient typically developed
SCFA	Short-chain fatty acids
SFARI	The Simons Foundation Autism Research Initiative
SNAP-IV	Swanson, Nolan, and Pelham-IV
Sp	Spore-forming bacteria
SPARK	Simons Foundation Powering Autism Research for Knowledge
SRS	Social Responsiveness Scale
StdADOS	Standardized Autism Diagnostic Observation Schedule
TD	Typically developed
TMAO	Trimethylamine-N-oxide
TMAV	N, N, N-trimethyl-5-aminovalerate
TXB2	Thromboxane B2
US	United States
VABS	Vineland Adaptive Behavior Scale
Vineland-3	Vineland Adaptive Behavior Scales, Third Edition
VPA	Valproic acid

## References

[1] Backhed F., Ley R.E., Sonnenburg J.L., Peterson D.A., Gordon J.I. (2005). Host-bacterial mutualism in the human intestine. Science.

[2] Mihailovich M., Sokovic Bajic S., Dinic M., Dokic J., Zivkovic M., Radojevic D., Golic N. (2024). Cutting-Edge iPSC-Based Approaches in Studying Host-Microbe Interactions in Neuropsychiatric Disorders. Int. J. Mol. Sci..

[3] Bojović K., Ignjatović Ð.D.I., Soković Bajić S., Vojnović Milutinović D., Tomić M., Golić N., Tolinački M. (2020). Gut Microbiota Dysbiosis Associated with Altered Production of Short Chain Fatty Acids in Children with Neurodevelopmental Disorders. Front. Cell. Infect. Microbiol..

[4] Hill C., Guarner F., Reid G., Gibson G.R., Merenstein D.J., Pot B., Morelli L., Canani R.B., Flint H.J., Salminen S. (2014). Expert consensus document. The International Scientific Association for Probiotics and Prebiotics consensus statement on the scope and appropriate use of the term probiotic. Nat. Rev. Gastroenterol. Hepatol..

[5] Soleimanpour S., Abavisani M., Khoshrou A., Sahebkar A. (2024). Probiotics for autism spectrum disorder: An updated systematic review and meta-analysis of effects on symptoms. J. Psychiatr. Res..

[6] Yang C., Xiao H., Zhu H., Du Y., Wang L. (2024). Revealing the gut microbiome mystery: A meta-analysis revealing differences between individuals with autism spectrum disorder and neurotypical children. Biosci. Trends.

[7] Mehra A., Arora G., Sahni G., Kaur M., Singh H., Singh B., Kaur S. (2023). Gut microbiota and Autism Spectrum Disorder: From pathogenesis to potential therapeutic perspectives. J. Tradit. Complement. Med..

[8] Zeng P., Zhang C.Z., Fan Z.X., Yang C.J., Cai W.Y., Huang Y.F., Xiang Z.J., Wu J.Y., Zhang J., Yang J. (2024). Effect of probiotics on children with autism spectrum disorders: A meta-analysis. Ital. J. Pediatr..

[9] He X., Liu W., Tang F., Chen X., Song G. (2023). Effects of Probiotics on Autism Spectrum Disorder in Children: A Systematic Review and Meta-Analysis of Clinical Trials. Nutrients.

[10] Feng P., Zhao S., Zhang Y., Li E. (2023). A review of probiotics in the treatment of autism spectrum disorders: Perspectives from the gut-brain axis. Front. Microbiol..

[11] Lord C., Brugha T.S., Charman T., Cusack J., Dumas G., Frazier T., Jones E.J.H., Jones R.M., Pickles A., State M.W. (2020). Autism spectrum disorder. Nat. Rev. Dis. Primers.

[12] Milovanovic M., Radivojevic V., Radosavljev-Kircanski J., Grujicic R., Toskovic O., Aleksic-Hil O., Pejovic-Milovancevic M. (2019). Epilepsy and interictal epileptiform activity in patients with autism spectrum disorders. Epilepsy Behav..

[13] Raleva M., Stancheva-Popkostadinova V., Pejovic-Milovancevic M. (2021). Editorial: Psychiatric Comorbidities in Children and Adolescents With ASD and in Typically Developing Children. Front. Psychiatry.

[14] Madra M., Ringel R., Margolis K.G. (2020). Gastrointestinal Issues and Autism Spectrum Disorder. Child. Adolesc. Psychiatr. Clin. N. Am..

[15] Kanner L. (1943). Autistic disturbances of affective contact. Nervous Child..

[16] Bresnahan M., Hornig M., Schultz A.F., Gunnes N., Hirtz D., Lie K.K., Magnus P., Reichborn-Kjennerud T., Roth C., Schjolberg S. (2015). Association of maternal report of infant and toddler gastrointestinal symptoms with autism: Evidence from a prospective birth cohort. JAMA Psychiatry.

[17] Wang L.W., Tancredi D.J., Thomas D.W. (2011). The prevalence of gastrointestinal problems in children across the United States with autism spectrum disorders from families with multiple affected members. J. Dev. Behav. Pediatr..

[18] Kang V., Wagner G.C., Ming X. (2014). Gastrointestinal dysfunction in children with autism spectrum disorders. Autism Res..

[19] Maenner M.J., Arneson C.L., Levy S.E., Kirby R.S., Nicholas J.S., Durkin M.S. (2012). Brief report: Association between behavioral features and gastrointestinal problems among children with autism spectrum disorder. J. Autism Dev. Disord..

[20] Chaidez V., Hansen R.L., Hertz-Picciotto I. (2014). Gastrointestinal problems in children with autism, developmental delays or typical development. J. Autism Dev. Disord..

[21] Buie T., Campbell D.B., Fuchs G.J., Furuta G.T., Levy J., Vandewater J., Whitaker A.H., Atkins D., Bauman M.L., Beaudet A.L. (2010). Evaluation, diagnosis, and treatment of gastrointestinal disorders in individuals with ASDs: A consensus report. Pediatrics.

[22] Folstein S., Rutter M. (1977). Genetic influences and infantile autism. Nature.

[23] Bailey A., Le Couteur A., Gottesman I., Bolton P., Simonoff E., Yuzda E., Rutter M. (1995). Autism as a strongly genetic disorder: Evidence from a British twin study. Psychol. Med..

[24] Huguet G., Benabou M., Bourgeron T., Sassone-Corsi P., Christen Y. (2016). The Genetics of Autism Spectrum Disorders. A Time for Metabolism and Hormones.

[25] Vicari S., Napoli E., Cordeddu V., Menghini D., Alesi V., Loddo S., Novelli A., Tartaglia M. (2019). Copy number variants in autism spectrum disorders. Prog. Neuropsychopharmacol. Biol. Psychiatry.

[26] Basilico B., Morandell J., Novarino G. (2020). Molecular mechanisms for targeted ASD treatments. Curr. Opin. Genet. Dev..

[27] Gaugler T., Klei L., Sanders S.J., Bodea C.A., Goldberg A.P., Lee A.B., Mahajan M., Manaa D., Pawitan Y., Reichert J. (2014). Most genetic risk for autism resides with common variation. Nat. Genet..

[28] Rolland T., Cliquet F., Anney R.J.L., Moreau C., Traut N., Mathieu A., Huguet G., Duan J., Warrier V., Portalier S. (2023). Phenotypic effects of genetic variants associated with autism. Nat. Med..

[29] Jones C., Barrera I., Brothers S., Ring R., Wahlestedt C. (2017). Oxytocin and social functioning. Dialogues Clin. Neurosci..

[30] Hollis F., Kanellopoulos A.K., Bagni C. (2017). Mitochondrial dysfunction in Autism Spectrum Disorder: Clinical features and perspectives. Curr. Opin. Neurobiol..

[31] Lalli M.A., Avey D., Dougherty J.D., Milbrandt J., Mitra R.D. (2020). High-throughput single-cell functional elucidation of neurodevelopmental disease-associated genes reveals convergent mechanisms altering neuronal differentiation. Genome Res..

[32] Lopez-Tobon A., Shyti R., Villa C.E., Cheroni C., Fuentes-Bravo P., Trattaro S., Caporale N., Troglio F., Tenderini E., Mihailovich M. (2023). GTF2I dosage regulates neuronal differentiation and social behavior in 7q11.23 neurodevelopmental disorders. Sci. Adv..

[33] Mihailovich M., Germain P.L., Shyti R., Pozzi D., Noberini R., Liu Y., Aprile D., Tenderini E., Troglio F., Trattaro S. (2024). Multiscale modeling uncovers 7q11.23 copy number variation-dependent changes in ribosomal biogenesis and neuronal maturation and excitability. J. Clin. Investig..

[34] Paulsen B., Velasco S., Kedaigle A.J., Pigoni M., Quadrato G., Deo A.J., Adiconis X., Uzquiano A., Sartore R., Yang S.M. (2022). Autism genes converge on asynchronous development of shared neuron classes. Nature.

[35] Wild C.P. (2005). Complementing the genome with an “exposome”: The outstanding challenge of environmental exposure measurement in molecular epidemiology. Cancer Epidemiol. Biomarkers Prev..

[36] Maitre L., de Bont J., Casas M., Robinson O., Aasvang G.M., Agier L., Andrusaityte S., Ballester F., Basagana X., Borras E. (2018). Human Early Life Exposome (HELIX) study: A European population-based exposome cohort. BMJ Open.

[37] McCanlies E.C., Ma C.C., Gu J.K., Fekedulegn D., Sanderson W.T., Ludena-Rodriguez Y.J., Hertz-Picciotto I. (2019). The CHARGE study: An assessment of parental occupational exposures and autism spectrum disorder. Occup. Environ. Med..

[38] Mandic-Maravic V., Mitkovic-Voncina M., Pljesa-Ercegovac M., Savic-Radojevic A., Djordjevic M., Pekmezovic T., Grujicic R., Ercegovac M., Simic T., Lecic-Tosevski D. (2019). Autism Spectrum Disorders and Perinatal Complications-Is Oxidative Stress the Connection?. Front. Psychiatry.

[39] Modabbernia A., Velthorst E., Reichenberg A. (2017). Environmental risk factors for autism: An evidence-based review of systematic reviews and meta-analyses. Mol. Autism.

[40] Yenkoyan K., Mkhitaryan M., Bjorklund G. (2024). Environmental Risk Factors in Autism Spectrum Disorder: A Narrative Review. Curr. Med. Chem..

[41] Mandic-Maravic V., Pljesa-Ercegovac M., Mitkovic-Voncina M., Savic-Radojevic A., Lecic-Tosevski D., Simic T., Pejovic-Milovancevic M. (2017). Impaired Redox Control in Autism Spectrum Disorders: Could It Be the X in GxE?. Curr. Psychiatry Rep..

[42] Miko E., Csaszar A., Bodis J., Kovacs K. (2022). The Maternal-Fetal Gut Microbiota Axis: Physiological Changes, Dietary Influence, and Modulation Possibilities. Life.

[43] Di Gesu C.M., Buffington S.A. (2024). The early life exposome and autism risk: A role for the maternal microbiome?. Gut Microbes.

[44] Hsiao E.Y., McBride S.W., Hsien S., Sharon G., Hyde E.R., McCue T., Codelli J.A., Chow J., Reisman S.E., Petrosino J.F. (2013). Microbiota modulate behavioral and physiological abnormalities associated with neurodevelopmental disorders. Cell.

[45] Malkova N.V., Yu C.Z., Hsiao E.Y., Moore M.J., Patterson P.H. (2012). Maternal immune activation yields offspring displaying mouse versions of the three core symptoms of autism. Brain Behav. Immun..

[46] Mazmanian S.K., Round J.L., Kasper D.L. (2008). A microbial symbiosis factor prevents intestinal inflammatory disease. Nature.

[47] Caetano-Silva M.E., Rund L., Hutchinson N.T., Woods J.A., Steelman A.J., Johnson R.W. (2023). Inhibition of inflammatory microglia by dietary fiber and short-chain fatty acids. Sci. Rep..

[48] Lin C.W., Septyaningtrias D.E., Chao H.W., Konda M., Atarashi K., Takeshita K., Tamada K., Nomura J., Sasagawa Y., Tanaka K. (2022). A common epigenetic mechanism across different cellular origins underlies systemic immune dysregulation in an idiopathic autism mouse model. Mol. Psychiatry.

[49] Meyza K.Z., Blanchard D.C. (2017). The BTBR mouse model of idiopathic autism—Current view on mechanisms. Neurosci. Biobehav. Rev..

[50] Sajdel-Sulkowska E.M. (2023). The Impact of Maternal Gut Microbiota during Pregnancy on Fetal Gut-Brain Axis Development and Life-Long Health Outcomes. Microorganisms.

[51] Kong L., Norstedt G., Schalling M., Gissler M., Lavebratt C. (2018). The Risk of Offspring Psychiatric Disorders in the Setting of Maternal Obesity and Diabetes. Pediatrics.

[52] Krakowiak P., Walker C.K., Bremer A.A., Baker A.S., Ozonoff S., Hansen R.L., Hertz-Picciotto I. (2012). Maternal metabolic conditions and risk for autism and other neurodevelopmental disorders. Pediatrics.

[53] Gardner R.M., Lee B.K., Magnusson C., Rai D., Frisell T., Karlsson H., Idring S., Dalman C. (2015). Maternal body mass index during early pregnancy, gestational weight gain, and risk of autism spectrum disorders: Results from a Swedish total population and discordant sibling study. Int. J. Epidemiol..

[54] Arentsen T., Raith H., Qian Y., Forssberg H., Diaz Heijtz R. (2015). Host microbiota modulates development of social preference in mice. Microb. Ecol. Health Dis..

[55] Bercik P., Denou E., Collins J., Jackson W., Lu J., Jury J., Deng Y., Blennerhassett P., Macri J., McCoy K.D. (2011). The intestinal microbiota affect central levels of brain-derived neurotropic factor and behavior in mice. Gastroenterology.

[56] Desbonnet L., Garrett L., Clarke G., Bienenstock J., Dinan T.G. (2008). The probiotic Bifidobacteria infantis: An assessment of potential antidepressant properties in the rat. J. Psychiatr. Res..

[57] Mohajeri M.H., La Fata G., Steinert R.E., Weber P. (2018). Relationship between the gut microbiome and brain function. Nutr. Rev..

[58] Neufeld K.M., Kang N., Bienenstock J., Foster J.A. (2011). Reduced anxiety-like behavior and central neurochemical change in germ-free mice. Neurogastroenterol. Motil..

[59] Sudo N., Chida Y., Aiba Y., Sonoda J., Oyama N., Yu X.N., Kubo C., Koga Y. (2004). Postnatal microbial colonization programs the hypothalamic-pituitary-adrenal system for stress response in mice. J. Physiol..

[60] Vuong H.E., Pronovost G.N., Williams D.W., Coley E.J.L., Siegler E.L., Qiu A., Kazantsev M., Wilson C.J., Rendon T., Hsiao E.Y. (2020). The maternal microbiome modulates fetal neurodevelopment in mice. Nature.

[61] Patterson P.H. (2011). Modeling autistic features in animals. Pediatr. Res..

[62] Xiao L., Yan J., Yang T., Zhu J., Li T., Wei H., Chen J. (2021). Fecal Microbiome Transplantation from Children with Autism Spectrum Disorder Modulates Tryptophan and Serotonergic Synapse Metabolism and Induces Altered Behaviors in Germ-Free Mice. mSystems.

[63] Sharon G., Cruz N.J., Kang D.W., Gandal M.J., Wang B., Kim Y.M., Zink E.M., Casey C.P., Taylor B.C., Lane C.J. (2019). Human Gut Microbiota from Autism Spectrum Disorder Promote Behavioral Symptoms in Mice. Cell.

[64] Nicolini C., Fahnestock M. (2018). The valproic acid-induced rodent model of autism. Exp. Neurol..

[65] Franzosa E.A., McIver L.J., Rahnavard G., Thompson L.R., Schirmer M., Weingart G., Lipson K.S., Knight R., Caporaso J.G., Segata N. (2018). Species-level functional profiling metagenomes and metatranscriptomes. Nat. Methods.

[66] Noecker C., Eng A., Muller E., Borenstein E. (2022). MIMOSA2: A metabolic network-based tool for inferring mechanism-supported relationships in microbiome-metabolome of data. Bioinformatics.

[67] Shah S., Molinaro G., Liu B., Wang R., Huber K.M., Richter J.D. (2020). FMRP Control of Ribosome Translocation Promotes Chromatin Modifications and Alternative Splicing of Neuronal Genes Linked to Autism. Cell Rep..

[68] Gandal M.J., Zhang P., Hadjimichael E., Walker R.L., Chen C., Liu S., Won H., van Bakel H., Varghese M., Wang Y. (2018). Transcriptome-wide isoform-level dysregulation in ASD, schizophrenia, and bipolar disorder. Science.

[69] Dutta D.J., Sasaki J., Bansal A., Sugai K., Yamashita S., Li G., Lazarski C., Wang L., Sasaki T., Yamashita C. (2023). Alternative splicing events as peripheral biomarkers for motor learning deficit caused by adverse prenatal environments. Proc. Natl. Acad. Sci. USA.

[70] Shah S., Sharp K.J., Raju Ponny S., Lee J., Watts J.K., Berry-Kravis E., Richter J.D. (2023). Antisense oligonucleotide rescue of CGG expansion-dependent FMR1 mis-splicing in fragile X syndrome restores FMRP. Proc. Natl. Acad. Sci. USA.

[71] Ghodke-Puranik Y., Thorn C.F., Lamba J.K., Leeder J.S., Song W., Birnbaum A.K., Altman R.B., Klein T.E. (2013). Valproic acid pathway: Pharmacokinetics and pharmacodynamics. Pharmacogenet Genom..

[72] Payne M., Mali I., McKinnell Z.E., Vangsness L., Shrestha T.B., Bossmann S.H., Plakke B. (2021). Increased volumes of lobule VI in a valproic acid model of autism are associated with worse set-shifting performance in male Long-Evan rats. Brain Res..

[73] Taleb A., Lin W., Xu X., Zhang G., Zhou Q.G., Naveed M., Meng F., Fukunaga K., Han F. (2021). Emerging mechanisms of valproic acid-induced neurotoxic events in autism and its implications for pharmacological treatment. Biomed. Pharmacother..

[74] De Angelis M., Piccolo M., Vannini L., Siragusa S., De Giacomo A., Serrazzanetti D.I., Cristofori F., Guerzoni M.E., Gobbetti M., Francavilla R. (2013). Fecal microbiota and metabolome of children with autism and pervasive developmental disorder not otherwise specified. PLoS ONE.

[75] Kang D.W., Ilhan Z.E., Isern N.G., Hoyt D.W., Howsmon D.P., Shaffer M., Lozupone C.A., Hahn J., Adams J.B., Krajmalnik-Brown R. (2018). Differences in fecal microbial metabolites and microbiota of children with autism spectrum disorders. Anaerobe.

[76] Hepsomali P., Groeger J.A., Nishihira J., Scholey A. (2020). Effects of Oral Gamma-Aminobutyric Acid (GABA) Administration on Stress and Sleep in Humans: A Systematic Review. Front. Neurosci..

[77] Braga J.D., Thongngam M., Kumrungsee T. (2024). Gamma-aminobutyric acid as a potential postbiotic mediator in the gut-brain axis. NPJ Sci. Food.

[78] Sokovic Bajic S., Djokic J., Dinic M., Veljovic K., Golic N., Mihajlovic S., Tolinacki M. (2019). GABA-Producing Natural Dairy Isolate From Artisanal Zlatar Cheese Attenuates Gut Inflammation and Strengthens Gut Epithelial Barrier in vitro. Front. Microbiol..

[79] Altaib H., Nakamura K., Abe M., Badr Y., Yanase E., Nomura I., Suzuki T. (2021). Differences in the Concentration of the Fecal Neurotransmitters GABA and Glutamate Are Associated with Microbial Composition among Healthy Human Subjects. Microorganisms.

[80] Erickson C.A., Wink L.K., Ray B., Early M.C., Stiegelmeyer E., Mathieu-Frasier L., Patrick V., Lahiri D.K., McDougle C.J. (2013). Impact of acamprosate on behavior and brain-derived neurotrophic factor: An open-label study in youth with fragile X syndrome. Psychopharmacology.

[81] Jiang S., Xiao L., Sun Y., He M., Gao C., Zhu C., Chang H., Ding J., Li W., Wang Y. (2022). The GABAB receptor agonist STX209 reverses the autism--like behaviour in an animal model of autism induced by prenatal exposure to valproic acid. Mol. Med. Rep..

[82] Donaldson Z.R., Young L.J. (2008). Oxytocin, vasopressin, and the neurogenetics of sociality. Science.

[83] Okumura T., Nozu T., Ishioh M., Igarashi S., Funayama T., Kumei S., Ohhira M. (2022). Oxytocin acts centrally in the brain to improve leaky gut through the vagus nerve and a cannabinoid signaling in rats. Physiol. Behav..

[84] Poutahidis T., Kearney S.M., Levkovich T., Qi P., Varian B.J., Lakritz J.R., Ibrahim Y.M., Chatzigiagkos A., Alm E.J., Erdman S.E. (2013). Microbial symbionts accelerate wound healing via the neuropeptide hormone oxytocin. PLoS ONE.

[85] Buffington S.A., Di Prisco G.V., Auchtung T.A., Ajami N.J., Petrosino J.F., Costa-Mattioli M. (2016). Microbial Reconstitution Reverses Maternal Diet-Induced Social and Synaptic Deficits in Offspring. Cell.

[86] Mazzone L., Dooling S.W., Volpe E., Uljarevic M., Waters J.L., Sabatini A., Arturi L., Abate R., Riccioni A., Siracusano M. (2024). Precision microbial intervention improves social behavior but not autism severity: A pilot double-blind randomized placebo-controlled trial. Cell Host Microbe.

[87] Schmitt L.M., Smith E.G., Pedapati E.V., Horn P.S., Will M., Lamy M., Barber L., Trebley J., Meyer K., Heiman M. (2023). Results of a phase Ib study of SB-121, an investigational probiotic formulation, a randomized controlled trial in participants with autism spectrum disorder. Sci. Rep..

[88] Billeci L., Callara A.L., Guiducci L., Prosperi M., Morales M.A., Calderoni S., Muratori F., Santocchi E. (2023). A randomized controlled trial into the effects of probiotics on electroencephalography in preschoolers with autism. Autism.

[89] Niu M., Li Q., Zhang J., Wen F., Dang W., Duan G., Li H., Ruan W., Yang P., Guan C. (2019). Characterization of Intestinal Microbiota and Probiotics Treatment in Children With Autism Spectrum Disorders in China. Front. Neurol..

[90] Meguid N.A., Mawgoud Y.I.A., Bjørklund G., Mehanne N.S., Anwar M., Effat B.A.E.K., Chirumbolo S., Elrahman M.M.A. (2022). Molecular Characterization of Probiotics and Their Influence on Children with Autism Spectrum Disorder. Mol. Neurobiol..

[91] Li Y.Q., Sun Y.H., Liang Y.P., Zhou F., Yang J., Jin S.L. (2021). Effect of probiotics combined with applied behavior analysis in the treatment of children with autism spectrum disorder: A prospective randomized controlled trial. Chin. J. Contemp. Pediatr..

[92] Guidetti C., Salvini E., Viri M., Deidda F., Amoruso A., Visciglia A., Drago L., Calgaro M., Vitulo N., Pane M. (2022). Randomized Double-Blind Crossover Study for Evaluating a Probiotic Mixture on Gastrointestinal and Behavioral Symptoms of Autistic Children. J. Clin. Med..

[93] Liu Y.W., Liong M.T., Chung Y.C.E., Huang H.Y., Peng W.S., Cheng Y.F., Lin Y.S., Wu Y.Y., Tsai Y.C. (2019). Effects of Lactobacillus plantarum PS128 on Children with Autism Spectrum Disorder in Taiwan: A Randomized, Double-Blind, Placebo-Controlled Trial. Nutrients.

[94] Kong X.J., Liu J., Liu K., Koh M., Sherman H., Liu S., Tian R., Sukijthamapan P., Wang J., Fong M. (2021). Probiotic and Oxytocin Combination Therapy in Patients with Autism Spectrum Disorder: A Randomized, Double-Blinded, Placebo-Controlled Pilot Trial. Nutrients.

[95] Wang Y., Li N., Yang J.J., Zhao D.M., Chen B., Zhang G.Q., Chen S., Cao R.F., Yu H., Zhao C.Y. (2020). Probiotics and fructo-oligosaccharide intervention modulate the microbiota-gut brain axis to improve autism spectrum reducing also the hyper-serotonergic state and the dopamine metabolism disorder. Pharmacol. Res..

[96] Santocchi E., Guiducci L., Prosperi M., Calderoni S., Gaggini M., Apicella F., Tancredi R., Billeci L., Mastromarino P., Grossi E. (2020). Effects of Probiotic Supplementation on Gastrointestinal, Sensory and Core Symptoms in Autism Spectrum Disorders: A Randomized Controlled Trial. Front. Psychiatry.

[97] Arnold L.E., Luna R.A., Williams K., Chan J., Parker R.A., Wu Q., Hollway J.A., Jeffs A., Lu F., Coury D.L. (2019). Probiotics for Gastrointestinal Symptoms and Quality of Life in Autism: A Placebo-Controlled Pilot Trial. J. Child. Adolesc. Psychopharmacol..

[98] Parracho H.M., Gibson G.R., Knott F., Bosscher D., Kleerebezem M., McCartney A.L. (2010). A double-blind, placebo-controlled, crossover-designed probiotic feeding study in children diagnosed with autistic spectrum disorders. Int. J. Probiotics Prebiotics.

[99] Sanctuary M.R., Kain J.N., Chen S.Y., Kalanetra K., Lemay D.G., Rose D.R., Yang H.T., Tancredi D.J., German J.B., Slupsky C.M. (2019). Pilot study of probiotic/colostrum supplementation on gut function in children with autism and gastrointestinal symptoms. PLoS ONE.

[100] Shaaban S.Y., El Gendy Y.G., Mehanna N.S., El-Senousy W.M., El-Feki H.S., Saad K., El-Asheer O.M. (2018). The role of probiotics in children with autism spectrum disorder: A prospective, open-label study. Nutr. Neurosci..

[101] Adams J.B., Johansen L.J., Powell L.D., Quig D., Rubin R.A. (2011). Gastrointestinal flora and gastrointestinal status in children with autism--comparisons to typical children and correlation with autism severity. BMC Gastroenterol..

[102] EI-Alfy M.S.E., Youssef A., Sabrey R. (2019). A Study on Effect of Probiotic Supplementation on Gastrointesinal Symptomes, Cognition and Behavior in Egyptian Children with Autism Spectrum Disorder. Egypt. J. Pediatr..

[103] Kałużna-Czaplińska J., Błaszczyk S. (2012). The level of arabinitol in autistic children after probiotic therapy. Nutrition.

[104] West R., Roberts E., Sichel L.S., Sichel J. (2013). Improvements in Gastrointestinal Symptoms among Children with Autism Spectrum Disorder Receiving the Delpro^®^ Probiotic and Immunomodulator Formulation. J. Probiotics Health.

[105] Tomova A., Husarova V., Lakatosova S., Bakos J., Vlkova B., Babinska K., Ostatnikova D. (2015). Gastrointestinal microbiota in children with autism in Slovakia. Physiol. Behav..

[106] Tharawadeephimuk W., Chaiyasut C., Sirilun S., Sittiprapaporn P. Preliminary Study of Probiotics and Kynurenine Pathway in Autism Spectrum Disorder. Proceedings of the 16th International Conference on Electrical Engineering/Electronics, Computer, Telecommunications and Information Technology (ECTI-CON).

[107] Mensi M.M., Rogantini C., Marchesi M., Borgatti R., Chiappedi M. (2021). Lactobacillus plantarum PS128 and Other Probiotics in Children and Adolescents with Autism Spectrum Disorder: A Real-World Experience. Nutrients.

[108] Grossi E., Melli S., Dunca D., Terruzzi V. (2016). Unexpected improvement in core autism spectrum disorder symptoms after long-term treatment with probiotics. SAGE Open Med. Case Rep..

[109] Kobliner V., Mumper E., Baker S.M. (2018). Reduction in Obsessive Compulsive Disorder and Self-Injurious Behavior With Saccharomyces boulardii in a Child with Autism: A Case Report. Integr. Med..

[110] Liubakka A., Vaughn B.P. (2016). Clostridium difficile Infection and Fecal Microbiota Transplant. AACN Adv. Crit. Care.

[111] Kang D.W., Adams J.B., Gregory A.C., Borody T., Chittick L., Fasano A., Khoruts A., Geis E., Maldonado J., McDonough-Means S. (2017). Microbiota Transfer Therapy alters gut ecosystem and improves gastrointestinal and autism symptoms: An open-label study. Microbiome.

[112] Kang D.W., Adams J.B., Coleman D.M., Pollard E.L., Maldonado J., McDonough-Means S., Caporaso J.G., Krajmalnik-Brown R. (2019). Long-term benefit of Microbiota Transfer Therapy on autism symptoms and gut microbiota. Sci. Rep..

[113] Park S.Y., Seo G.S. (2021). Fecal Microbiota Transplantation: Is It Safe?. Clin. Endosc..

[114] Wilson B.C., Vatanen T., Cutfield W.S., O’Sullivan J.M. (2019). The Super-Donor Phenomenon in Fecal Microbiota Transplantation. Front. Cell Infect. Microbiol..

